# Soil Infiltration Characteristics in Agroforestry Systems and Their Relationships with the Temporal Distribution of Rainfall on the Loess Plateau in China

**DOI:** 10.1371/journal.pone.0124767

**Published:** 2015-04-20

**Authors:** Lai Wang, Chonggao Zhong, Pengxiang Gao, Weimin Xi, Shuoxin Zhang

**Affiliations:** 1 College of Forestry, Northwest A&F University, Yangling, Shaanxi, China; 2 Qingling National Forest Ecosystem Research Station, Yangling, Shaanxi, China; 3 Department of Biological and Health Sciences, Texas A&M University-Kingsville, Kingsville, Texas, United States of America; Murdoch University, AUSTRALIA

## Abstract

Many previous studies have shown that land use patterns are the main factors influencing soil infiltration. Thus, increasing soil infiltration and reducing runoff are crucial for soil and water conservation, especially in semi-arid environments. To explore the effects of agroforestry systems on soil infiltration and associated properties in a semi-arid area of the Loess Plateau in China, we compared three plant systems: a walnut (*Juglans regia*) monoculture system (JRMS), a wheat (*Triticum aestivum*) monoculture system (TAMS), and a walnut-wheat alley cropping system (JTACS) over a period of 11 years. Our results showed that the JTACS facilitated infiltration, and its infiltration rate temporal distribution showed a stronger relationship coupled with the rainfall temporal distribution compared with the two monoculture systems during the growing season. However, the effect of JTACS on the infiltration capacity was only significant in shallow soil layer, i.e., the 0–40 cm soil depth. Within JTACS, the speed of the wetting front’s downward movement was significantly faster than that in the two monoculture systems when the amount of rainfall and its intensity were higher. The soil infiltration rate was improved, and the two peaks of soil infiltration rate temporal distribution and the rainfall temporal distribution coupled in rainy season in the alley cropping system, which has an important significance in soil and water conservation. The results of this empirical study provide new insights into the sustainability of agroforestry, which may help farmers select rational planting patterns in this region, as well as other regions with similar climatic and environmental characteristics throughout the world.

## Introduction

In both arid and semi-arid areas, soil infiltration is recognized as a fundamental ecological process that affects the water budget of vegetation, runoff, and the related risk of soil erosion [1, 2]. Thus, permeability is an important indicator of soil erosion resistance [3]. Runoff and soil infiltration are important determinants of vegetation patterns [4], but vegetation patterns also modify the infiltration capacity directly [5, 6]. Therefore, reasonable land use patterns could improve the physical and chemical properties of soil, ameliorate the soil infiltration capacity, and influence soil water redistribution processes [7]. Water shortages and soil erosion are limiting factors that affect ecological recovery and agricultural production on the Loess Plateau in China [8]. Thus, maximizing the retention of precipitation resources and reducing runoff are crucial for the region’s ecological restoration and for agricultural production in this region [9].

Agroforestry management has been widely applied to reduce soil erosion and water losses, as well as to increase the land utilization rate and yield economic benefits [10, 11], thereby generating more products and increasing the incomes of farmers compared with traditional farming and forestry on the Loess Plateau [12]. The economic benefits of agroforestry as a land use management system mean that the ecological rationality and sustainability of this approach are often ignored. However, an unreasonable agroforestry model could reduce the soil infiltration capacity, increase the amount of evapotranspiration, or both, thereby causing soil desiccation and unsustainable farming practices [13, 14]. Previous studies of agroforestry practices have focused mainly on yields, rural income, interspecific competition, and complementarity, e.g., aboveground competition for light energy [15–17], underground competition for water and nutrients [18–20], competition for ecological plasticity in the roots [21–23], and the impact of these competitive factors on the yield and income for farmers [24–26]. However, few studies have addressed these competitive processes as a whole from the perspectives of ecological rationality and sustainability.

The present study was performed in the semi-arid loess area of the northern Wei River on the Loess Plateau, China. A widely practiced local agroforestry model, i.e., a walnut (*Juglans regia*)-wheat (*Triticum aestivum*) alley cropping system (JTACS), was our research focus, while the walnut (JRMS) and wheat (TAMS) monoculture systems were used as controls. The double ring infiltration method was used to determine the soil infiltration rate [27]. The effect of the agroforestry system on soil infiltration was studied for a period of 11 years to determine the regularity of infiltration and its relationship with the rainfall temporal distribution, where we tested the following hypotheses: 1) soil infiltration increased with the increased of intercropping age in the agroforestry system, and 2) seasonal variation in infiltration in the agroforestry system was related to the rainfall temporal distribution of in the region. Our results may facilitate the development of strategies for soil and water conservation in the context of agricultural sustainability on the Loess Plateau, as well as other areas with similar soil erosion problems.

## Materials and Methods

### Ethics Statement

The study was carried out on collective land, and we confirm that the owner of the land (Weiweiyuan Walnut Professional Farmer Cooperatives) gave permission to conduct the study on these site. We also confirm that the field studies did not involve endangered or protected species.

### Study area

The study site was located in Zhangjiagou Village, Qishan County, Shaanxi Province, China (107°43′42″E, 34°21′21″N). The site’s elevation is 736 m and the north-facing terrain has a slope of 2–15°. Various types of agroforestry systems cover an area of about 130 ha, but the most common one is tree alley cropping systems, which account for about 90%. The mean annual rainfall in this area is 672 mm, where about 70% falls in 6–9 months, and the aridity index is 1.1. The mean annual sunshine duration is 2053.3 h and the frost-free period is 215 days. The absolute minimum temperature is −20.6°C, the maximum temperature is 41.4°C, the annual mean temperature is 12.0°C, and the ≥ 10°C accumulated temperature is 3826°C. The depth of the phreatic water table is 80–120 m and the soil is 100–250 m deep. The tillage layer is cinnamon soil, developed from the loess parent material, with an organic content of 16.2 g kg^–1^ and available nitrogen, phosphorus, and potassium contents of 97.4, 18.6, and 61.3 mg kg^–1^, respectively.

### Experimental design

The experimental field is comprised of a block of terraces in the middle of hills, which were constructed in the mid-1970s. Along the contour, the field is about 300 m long and 30–45 m wide, with a total size of area was 1.1 ha. The slope aspect was toward the north and the degree was 2–3°. The soil texture in the tillage layer was clay loam. The field was designed as a long-term permanent sample plot in 2003, where it was planted with JTACS, JRMS, and TAMS from east to west, with planting areas of 0.5, 0.4, and 0.2 ha, respectively.

Wheat was planted in one season and the field was left fallow in the summer and fall in a year. Conventional tillage was applied before planting wheat in the following year. The sowing and harvest dates for winter wheat were about October 5 and June 5. The walnut tree plantings in JRMS and JTACS had the same staggered arrangement. Clean tillage was implemented for JRMS. In JTACS, a 1-m control belt was set aside for the line of walnut trees, where the wheat planting width was 5 m. The specific wheat plantings in JTACS and TAMS had the same arrangement (Table 1).

**Table 1:** Planting arrangement and characteristics of the experimental site.

Experimental material	Cultivated variety	Row direction	Row spacing	Basic seeding rate	DBH	Height	Canopy
Wheat	Xiaoyan 22	North-south	20 cm	2.1 × 10^6^ ha^-1^			
Walnut	Xiangling	North-south	6 m	556 ha^-1^	13.2 cm	4.3 m	3 × 2.6 m

Note: Characteristics of walnut trees at the experimental site during July 2013 (11years). DBH: diameter at breast height.

Double ring infiltration measurement points were installed as follows: the diameter at breast height, height and width of canopy of all trees in a sample plot were measured. Then, we selected trees that the three indicators are all closed to the mean, respectively, these trees are called average trees. Three average trees were selected in JTACS, where the distribution of the average trees avoided the edges of the sample plot. Three measurement points were set for each average tree at distances of 1, 2, and 3 m perpendicular to the direction of the tree line. The arrangement of the measurement points was the same in JRMS and JTACS. In TAMS, the arrangement of the measurement points followed the classic five-point sampling method.

Since 2003, the soil infiltration rate in each sample plot was measured at the middle of August every other year. The soil infiltration rate was also measured in the middle of each month from April to October during 2011 and 2013. The infiltration rate at different soil depths was measured by removing the soil layer by layer starting from the surface in the middle of August 2013.

### Measurements

The classic double-ring method was used to determinate the soil infiltration rate [27]. The rings measured 18.5 cm in height, and the diameters of the outer and inner rings were 50 cm and 35 cm, respectively. The rings were inserted 12.5 cm into the soil and both were filled with water using two Mariotte bottles. Measurements were obtained from the inner ring when the water head was approximately 3 cm. The water level readings were determined from the bottle that supplied water to the inner ring at 0.0, 0.5, 1, 2, 3, 5, 7, 10, 15, 20, 25, and 30 min, and then at 10 min intervals subsequently. The water head was maintained at 3 cm in all of the rings during the experiment. The initial infiltration rate was considered to the average infiltration rate in the first 3 min. A constant infiltration rate was assumed to have been reached when five similar consecutive measurements were obtained [28]. Before measuring soil infiltration rate each time, we observed the soil water content of sampling points in 10 cm depth by soil moisture sensors. The soil water content was controlled within the range of 18±2% (V/V).

A multi-channel soil moisture recorder was placed perpendicular to the direction of the tree line and at a distance of 2 m from the average tree in JTACS and JRMS. In TAMS, three recorders were positioned randomly, where the edges of the sample plot were avoided. Soil moisture data were recorded once every 30 min and long-term continuous recording was initiated from June 2012. Soil moisture sensors (This is a kind of special soil moisture sensors: Product Model: ARN-100. Made by Hebei Aornor Electronic Technology Co., Ltd, China. Resolution is 0.1% (V/V)) were placed at soil depths of 10, 20, 40, 70, 110, 160, and 210 cm, at each observation point. The Soil water content reading of sensors will increase when the wetting front reaches the soil layer that contains the sensors. Time required from onset of rain to the readings increase was calculated, which was the time required for the wetting front to reach the soil layer. Two automatic precipitation recorders were placed in the experimental field to record rainfall, which were accurate within 0.1 mm. The rainfall distribution data were obtained from the local weather bureau.

Analysis of variance (ANOVA) and multiple comparisons (LSD), and repeated measures analyses were performed using the SAS v. 9.2 for Windows 7. Mean values were compared and significant differences were accepted at *P* < 0.05. Figures were produced using OriginPro 8.0 software. The research was conducted based on the Forestry Standards “Observation Methodology for Long-term Forest Ecosystem Research” in the People’s Republic of China [29].

## Results

### Infiltration rates variability during the growing season

**Fig 1:**
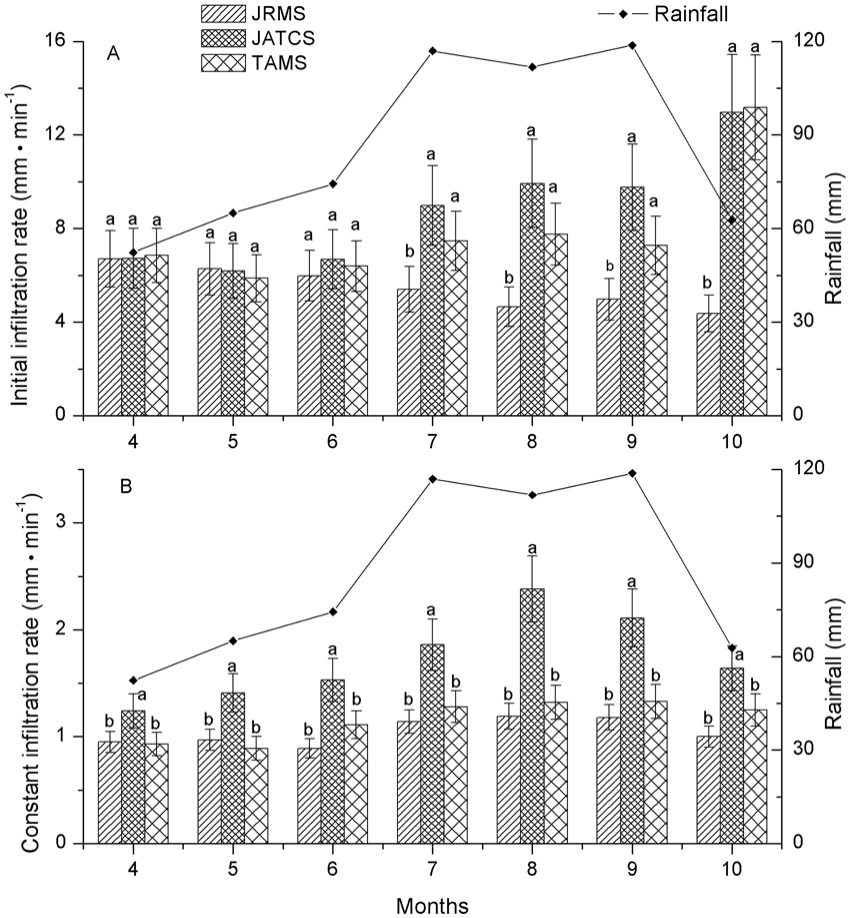
Characteristics of the infiltration rate during the growing season and its relationship with the rainfall temporal distribution. Note: JTCAS: walnut (*Juglans regia*)-wheat (*Triticum aestivum*) alley cropping system; JRMS: walnut monoculture system; TAMS: wheat monoculture system. Different lowercase letters at the top of the column plot indicate significant differences (P < 0.05).

During the growing season, the infiltration rates temporal distribution in JTACS all have a peak between July and September, and the rainfall temporal distribution in the region also have a peak in this period, the two peaks coupled (in October, the initial infiltration rate increased significantly because the land was plowed before sowing the winter wheat). This trend performed weaker in TAMS compared with JTACS. In JRMS, the trend performed the weakest among three land use patterns, even, there was a negative correlation between the initial infiltration rate distribution and the rainfall distribution (Fig 1).

The precipitation concentrated mainly in July, August, and September. In these three months, the initial infiltration rate was significantly higher in JTACS compared with that in JRMS (ANOVA: F = 5.31, *P* = 0.0471; F = 10.49, *P* = 0.0110; and F = 8.98, *P* = 0.0157; in July, August, and September, respectively), but no significant differences were observed in TAMS. In these three months, the average initial infiltration rate in JTACS was 9.56 mm·min^–1^, which was 1.91 and 1.27 times higher than that in JRMS and TAMS, respectively.

The constant infiltration rate was significantly higher in JTACS compared with that in JRMS and TAMS (ANOVA: F = 5.74, *P* = 0.0404; F = 12.95, *P* = 0.0067; F = 14.64, *P* = 0.0049; F = 14.23, *P* = 0.0053; F = 28.18, *P* = 0.0009; F = 18.97, *P* = 0.0023; F = 12.22, *P* = 0.0077; in each month from April to October, respectively), whereas there were no significant differences between JRMS and TAMS during the growing season. From July to September, the average constant infiltration rate in JTACS was 2.12 mm·min^–1^, which was 1.81 and 1.62 times higher than that in JRMS and TAMS, respectively.

### Interannual variability of infiltration rates

**Fig 2:**
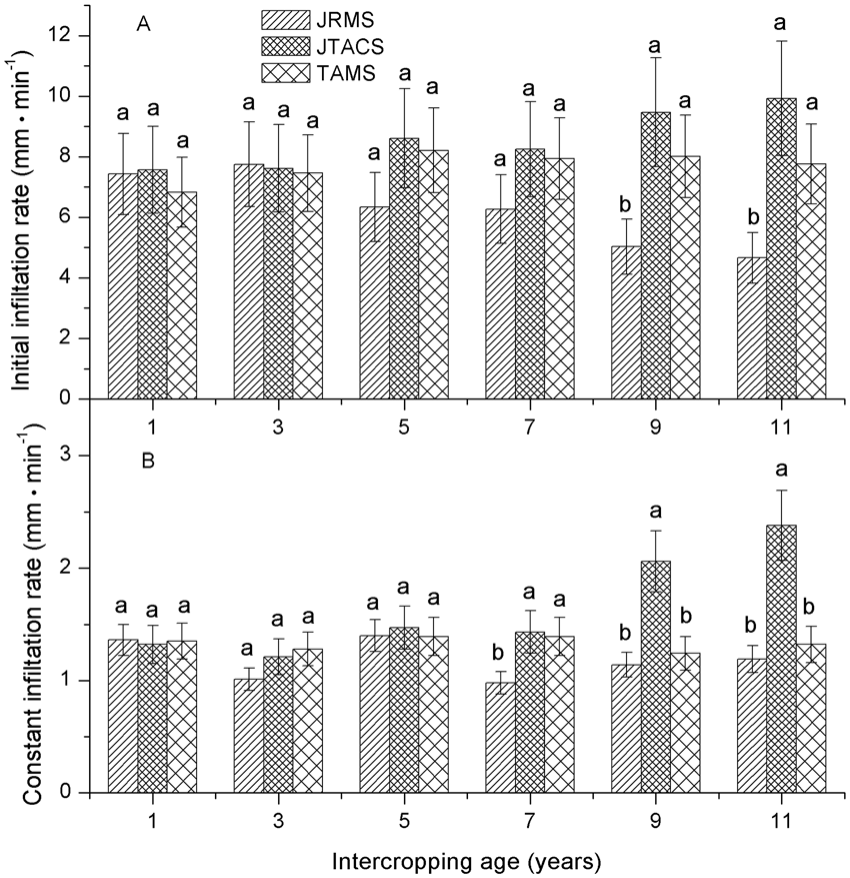
Characteristics of the interannual variation of the infiltration rate. Note: different lowercase letters at the top of the column plot indicate significant differences (P < 0.05).

In JTACS, the infiltration rates increased as the intercropping age increased. In TAMS, with the cropping age increased infiltration rates did not differ significantly. In JRMS, with the planting age increased, the initial infiltration rate tended to decrease gradually, whereas the constant infiltration rate did not differ significantly (Fig 2).

After 5 years, the initial infiltration rate in JRMS was less than that in JTACS. This difference increased with the intercropping age, whereas the difference was significant after 9 years (F = 7.79, *P* = 0.0215). The results of a repeated measures analysis showed that the initial infiltration rate in JTACS varied at different intercropping ages, whereas it was significantly higher after 9 years compared with that after 1 year. However, the opposite was found in JRMS. In JTACS, the initial infiltration rate was 9.93 mm min^–1^ after 11 years, which was 2.13 and 1.28 times higher than that in JRMS and TAMS, respectively.

In JTACS and JRMS, the difference in the constant infiltration rate was significant after 7 years, while the difference between JTACS and TAMS was significant after 9 years. The results of a repeated measures analysis showed that the constant infiltration rate in JTACS increased significant after 9 years. After 11 years, the constant infiltration rate was 2.38 mm·min^–1^ in JTACS, which was 2.0 and 1.8 times higher than that in JRMS and TAMS, respectively. In general, the initial infiltration rate and constant infiltration rate of JTACS all could be higher than the two monoculture systems significantly after 9 years.

### Infiltration rates at different soil depths

**Fig 3:**
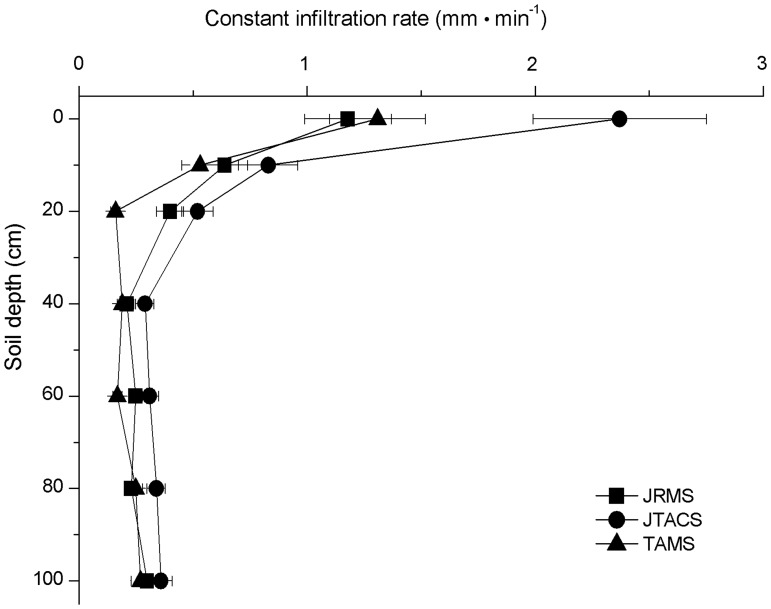
Variation of the infiltration rate as the soil depth increased.

As the soil depth increased, the constant infiltration rates declined rapidly in all three land use patterns, whereas the minimum infiltration rate occurred at depths of 20 cm in TAMS, and in JTACS and JRMS the depths are all at 40 cm, below which the infiltration rates were slightly higher in all three cases, while the average was generally 0.2–0.3 mm·min^–1^ (Fig 3).

The constant infiltration rate in JTACS was always significantly higher than that in JRMS and TAMS above the 40 cm soil layer, i.e., in the 0 cm (i.e., the earth’s surface) and 10 cm soil depths (ANOVA: F = 16.68, *P* = 0.0035; F = 6.29, *P* = 0.0336, respectively). In the 20 cm layer, the rate was significantly lower in TAMS compared with those in JRMS and JTACS (ANOVA: F = 30.47, *P* = 0.0007). There were no significant differences among the three land use patterns below the 40 cm soil depth.

### Infiltration curve

Based on large numbers of field observations and measurements, Fang Zhengsan adjusted the Kostiakov infiltration model [30] to obtain the Fang Zhengsan infiltration model, which is more suitable for the Loess Plateau [31]. The model is as follows:

$$K_{t}\;=\;K+\frac{K_{1}}{t^{a}},$$ (1)

where K_t_ is the instantaneous infiltration rate, K is the constant infiltration rate, K_1_ is the infiltration coefficient, a is a constant term, and t is the infiltration time.

**Fig 4:**
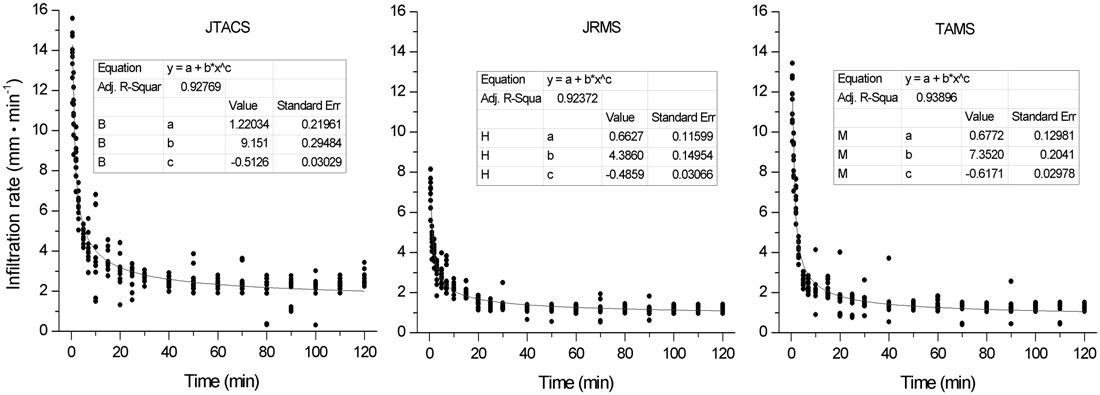
Soil infiltration curves for the three land use patterns in August 2013.

The infiltration curves fitted to the three land use patterns are shown in Fig 4. The equations fitted to the Fang Zhengsan infiltration model are as follows.

$$K_{t}\;=\;0.6627+4.3880t^{-0.4859}\;({\text{R}}^{2}\;=\;0.9237)\;(\text{JRMS})$$ (2)

$$K_{t}\;=\;1.2203+9.1510t^{-0.5126}\;{\text{(R}}^{2}\;=\;0.9277)\;(\text{JTACS})$$ (3)

$$K_{t}\;=\;0.6772+7.3520t^{-0.6171}\;{\text{(R}}^{2}\;=\;0.9390)\;(\text{TAMS})$$ (4)

According to the fitted equations, the infiltration coefficient and constant infiltration rates with the three land use patterns were in the order of: JTACS > TAMS > JRMS. The coefficients of variation for the infiltration rates in JTACS, TAMS, and JRMS were 25%, 22%, and 17%, respectively. This shows that the infiltration data of JTACS have more discreteness (Fig 4).

### Wetting front

During moderate rain, the time required of wetting front to reach each soil depth: TAMS was less than JTACS and JRMS significantly. The biggest infiltration depths of the three were all 40 cm (Table 2).

**Table 2:** Time required for the wetting front to reach specific soil depths with different rainfall levels (hours).

Daily rainfall (mm)	Land use pattern	Soil depth (cm)					
		10	20	40	70	110	160
19.7							
	JRMS	3.5±0.5^a^	10.0±0.5^c^	22.0±1.5^b^			
	TAMS	2.5±0.5^b^	6.0±0.5^d^	17.0±1.5^c^			
	JTACS	4.0±0.5^a^	9.0±1.0^c^	22.5±2.5^b^			
38.4							
	JRMS	2.0±0.5^bc^	4.0±1.5^d^	8.5±1.0^d^	28.5±3.5^c^	72.0±7.5^a^	296.5±24.5^a^
	TAMS	1.0±0.5^c^	2.5±0.5^e^	8.5±2.0^d^	36.5±3.5^b^	83.0±6.5^a^	319.5±21.5^a^
	JTACS	1.5±0.5^bc^	5.5±1.0^d^	8.0±1.5^d^	16.5±2.5^d^	57.5±5.5^b^	252.0±17.0^b^
73.1							
	JRMS	1.5±0.5^bc^	18.5±0.5^a^	27.5±1.5^a^			
	TAMS	1.0±0.5^c^	13.5±0.5^b^	25.0±1.5^ab^	45.0±3.5^a^		
	JTACS	1.5±0.5^bc^	10.5±0.5^c^	22.5±1.0^b^	36.5±2.5^b^	76.0±5.5^a^	

Note: Three rainfall events were observed during the observation period in 2013 (11 years), i.e., moderate rainfall of 19.7 mm on May 17, heavy rainfall of 38.4 mm on May 25, and torrential rainfall of 73.1 mm on July 17. Beginning when the rain started, the times required for the wetting front to reach different soil depths were recorded. The data represent the mean ± standard error. Different lowercase letters within a column indicate significant differences (*P* < 0.05). JTCAS: walnut (*Juglans regia*)-wheat (*Triticum aestivum*) alley cropping system; JRMS: walnut monoculture system; TAMS: wheat monoculture system.

During heavy rain, the time required for wetting front to reach 10, 20 cm soil depths: TAMS was less than JTACS and JRMS significantly, while the time to reach 40 cm soil depth no significant difference could be found among the three systems, whereas the time to reach below 40 cm soil depths: JTACS was less than TAMS and JRMS significantly. The biggest infiltration depths of the three were all 160 cm (Table 2).

During torrential rain, the time required for wetting front to reach 10 cm soil depth no significant difference could be observed among the three systems, whereas the time to reach 20 cm and below soil depths: JTACS was less than TAMS and JRMS significantly. Compared with TAMS and JRMS, JTACS had the biggest infiltration depths of 110 cm (Table 2).

In the same case, shorter time consumption means faster speed. So, in general, the wetting front moved downward in JTACS significantly faster compared with that in JRMS and TAMS during heavy and torrential rain. The wetting front in TAMS moved the fastest among the three systems during moderate rain. The speed of wetting front reached 10 cm soil depth in TAMS was the fastest among the three systems during moderate, heavy and torrential rain.

## Discussion

The results of this study support our hypothesis that the alley cropping system would increase the soil infiltration rate and enhance the relationship between soil infiltration rate temporal distribution and the rainfall temporal distribution. The experimental data used in this study were obtained from continuous observations at fixed sample plots in the field. We observed dynamic changes in the infiltration rate every other year and during the growing season, thereby ensuring the continuity and comparability of the data. The movements of the wetting fronts were determined in the 11-year-old fixed sample plots, which also supported our hypothesis from another aspect.

Cerdà reported that seasonal changes play important roles in soil hydrology [32], whereas the infiltration rate was the highest during the summer each year [33]. We obtained a similar result. However, Cerdà noted that the period with the maximum infiltration rate in hillside fields did not meet the local rainy season [33]. This may be attributable to differences regions and variations in the land use pattern. In the present study, the infiltration rate distribution in JRMS was also not coupled with the region’s rainfall distribution. In different land use patterns and cropping systems, plant root activities are important factors affecting soil infiltration [34, 35]. Thus, we speculated that main reasons for the peak of infiltration rate distribution in JTACS can meet with region’s rainy season in a year may be the result of the seasonal activity of plant roots and temperature seasonal changes. Wheat root rot in the shallow soil and walnut roots were active after the wheat harvest in June in JTACS. This makes the root channels more in soil and connectivity better, thereby the infiltration rate increased significantly from July. The increase in the infiltration rate meets with the region’s rainfall peak and there was a strong relationship. It is necessary to clear that the relationship between infiltration rate and rainfall distribution in a year just show the relevant, rather than the cause and effect.

As the age of the agroforestry systems increased, the accumulated influence of the roots may have changed the physical and chemical properties of the soil; thus, the soil infiltration capacity improved continuously. In TAMS, the seasonal variation in the constant infiltration rate was very low due to the lack of activity by deep walnut roots and because of the effect of the plow pan [36]. In JRMS, the surface soil became increasingly compact due to the lack of tillage and the activity of shallower wheat roots, which gradually reduced the infiltration rate [37].

During October, the initial infiltration rates were increased significantly by plowing in JTACS and TAMS, whereas their constant infiltration rates did not increase significantly. To some extent, our results also demonstrate that plowing can improve the initial infiltration rates significantly, but has no significant effect on the constant soil infiltration rate. The results reported by Matula [38].

Wu et al. reported that forest land could have a higher soil infiltration rate compared with wasteland, where the effective depth was about 40 cm [39], and a similar result was obtained in our study. This is consistent with the main activity area of tree roots (10–40 cm) in alley cropping systems [40]. At a depth of 20 cm, the infiltration rate was the lowest in TAMS among the three land use patterns and this difference may have been attributable to plow pan obstacles [41, 42]. We suggest that the activity of tree roots broke the plow pan obstacles in JTACS and JRMS, thereby promoting soil infiltration.

The wetting front moved downward faster in TAMS with lower rainfall, and in the shallow soil layer, compared with that in JTACS and JRMS. When the amount of rainfall was lower may be due to the existing canopy interception of tree in JTACS and JRMS, leading to the ground received less rain, finally its wetting front moved down slowly. The canopy interception ratio is negatively correlated with rainfall and its intensity [43]. When the amount of rainfall was higher, the canopy interception ratio was lower and it can even be neglected, that is to say, when the same rainfall received by ground among the different cropping systems, the fastest wetting front moving down speed was observed in JTACS. It also reflects the promoting for infiltration in the alley cropping system. Due to the increased infiltration rate, and retaining more water by wheat stubble [44], which are all can reduce surface runoff, so the wetting front’s speed of movement and the maximum infiltration depth were the biggest of JTACS in the three cropping systems. Thus, the soil and water conservation effects of JTACS were also highest when the amount of rainfall and its intensity were greater. These findings are highly significant for agricultural production and vegetation restoration on the Loess Plateau.

The main factors with direct effects on the soil infiltration capacity are the physical and chemical properties of soil. In the present study, we only considered changes in the infiltration process. However, the evolution of the physical and chemical properties of soil is complex, and thus long-term studies are necessary to understand this process.

## Conclusions

We found that the alley cropping system (JTACS) increased the soil infiltration rate compared with the monoculture systems. The effect of JTACS on the soil infiltration rate was significant after 9 years of intercropping, whereas the significant effect of JTACS on the soil infiltration capacity was detected in the 0–40 cm soil layer.

The speed of the wetting front’s downward movement and the maximum infiltration depth were bigger in the alley cropping system compared with those in the monoculture systems when the amount of rainfall was higher, which promoted water infiltration. This advantage declined or disappeared when the amount of rainfall was lower.

One of the most significant findings of this study was the two peaks of soil infiltration rate and rainfall temporal distribution coupled during the growing season in JTACS. This relationship has an important significance in soil and water conservation, especially in the semiarid environment. Rational land use patterns can enhance this relationship, such as JTACS, whereas irrational land use patterns will weaken the relationship, or even lead to a negative correlation, such as JRMS. The empirical findings of this study provide new insights into the sustainability of agroforestry on the Loess Plateau in China, as well as other regions with similar climatic and environmental characteristics throughout the world.
